# Diagnostic challenges in a CMMRD patient with a novel mutation in the *PMS2* gene: a case report

**DOI:** 10.1186/s12920-021-01031-9

**Published:** 2021-07-12

**Authors:** Shiqing Tan, Xiaoting Wu, Aoxue Wang, Li Ying

**Affiliations:** 1 Department of Gastroenterology, The Second Hospital of Dalian Medical University, Dalian, China; 2 Department of Dermatology, The Second Hospital of Dalian Medical University, Dalian, China; 3 The Third Clinical Medical College, Zhejiang Chinese Medical University, Hangzhou, China

**Keywords:** Constitutional mismatch repair deficiency (CMMRD), Glioma, Colorectal cancer, Café-au-lait macule, *PMS2* gene

## Abstract

**Background:**

Constitutional mismatch repair deficiency (CMMRD) is a rare autosomal recessive condition, which is caused by biallelic mutations in mismatch repair genes: *MSH2*, *MLH1*, *MSH6*, and *PMS2*.

**Case presentation:**

We reported a unique case of an 11-year-old Chinese girl with colorectal polyposis and café-au-lait macules who had no obvious family history of Lynch syndrome-associated tumors, followed by brain gliomas and colorectal carcinoma five years later. The diagnosis of CMMRD was based on gene sequencing analysis showing a homozygous deletion NM_00535.5:c.1577delA (p.Asp526fs) in exon 11 of the *PMS2* gene. Although the patient underwent surgery and radiation therapy, and close surveillances including radiological, endoscopic and hematological screening have been recommended, she died of the exacerbation of neurological symptoms at the age of 18.

**Conclusions:**

We identified a novel homozygous deletion in the *PMS2* gene in a CMMRD patient with complex clinical features.

**Supplementary Information:**

The online version contains supplementary material available at 10.1186/s12920-021-01031-9.

## Background

Mismatch repair (MMR) proteins can correct nucleotide mismatches during DNA replication process and act to maintain genomic stability. Defective MMR system results in the persistence of point mutation or frame-shift mutation and microsatellite instability (MSI), which drives neoplastic changes. Constitutional mismatch repair deficiency (CMMRD) is a rare MMR deficiency-related syndrome, resulting from a homozygous mutation in four MMR genes: *MSH2*, *MLH1*, *MSH6*, and *PMS2* [1]. This entity is characterized by a greater risk of developing childhood and adolescence malignancies, compared to Lynch syndrome (LS) which is caused by a heterozygous mutation in MMR genes and presents with cancers in adult life [1]. Brain malignant tumors are found in over 50% of all CMMRD patients, followed by gastrointestinal tract tumors and hematological tumors. Café-au-lait macules (CALMs) are the most common non-neoplastic manifestations of CMMRD. The prognosis of CMMRD patients is extremely poor due to aggressive phenotypes of tumors, with a median survival period of fewer than 30 months after the first diagnosis. Therefore, there is a clinical imperative to identify CMMRD because close surveillance is thought to greatly reduce cancer morbidity and mortality. Genetic testing for the MMR genes contributes to a confirmed diagnosis and can provide personalized management programs for both patients and their relatives. However, the diagnosis is often delayed because of obscure disease-specific clinical features. In some instances, it is even not stated owing to the lack of disease awareness. We herein presented a unique case of CMMRD who had CALMs and metachronous gliomas and colorectal cancer (CRC). The diagnosis was established by gene sequencing analysis showing a novel homozygous mutation (p.Asp526fs) in *PMS2*.

## Case presentation

**Fig. 1:**
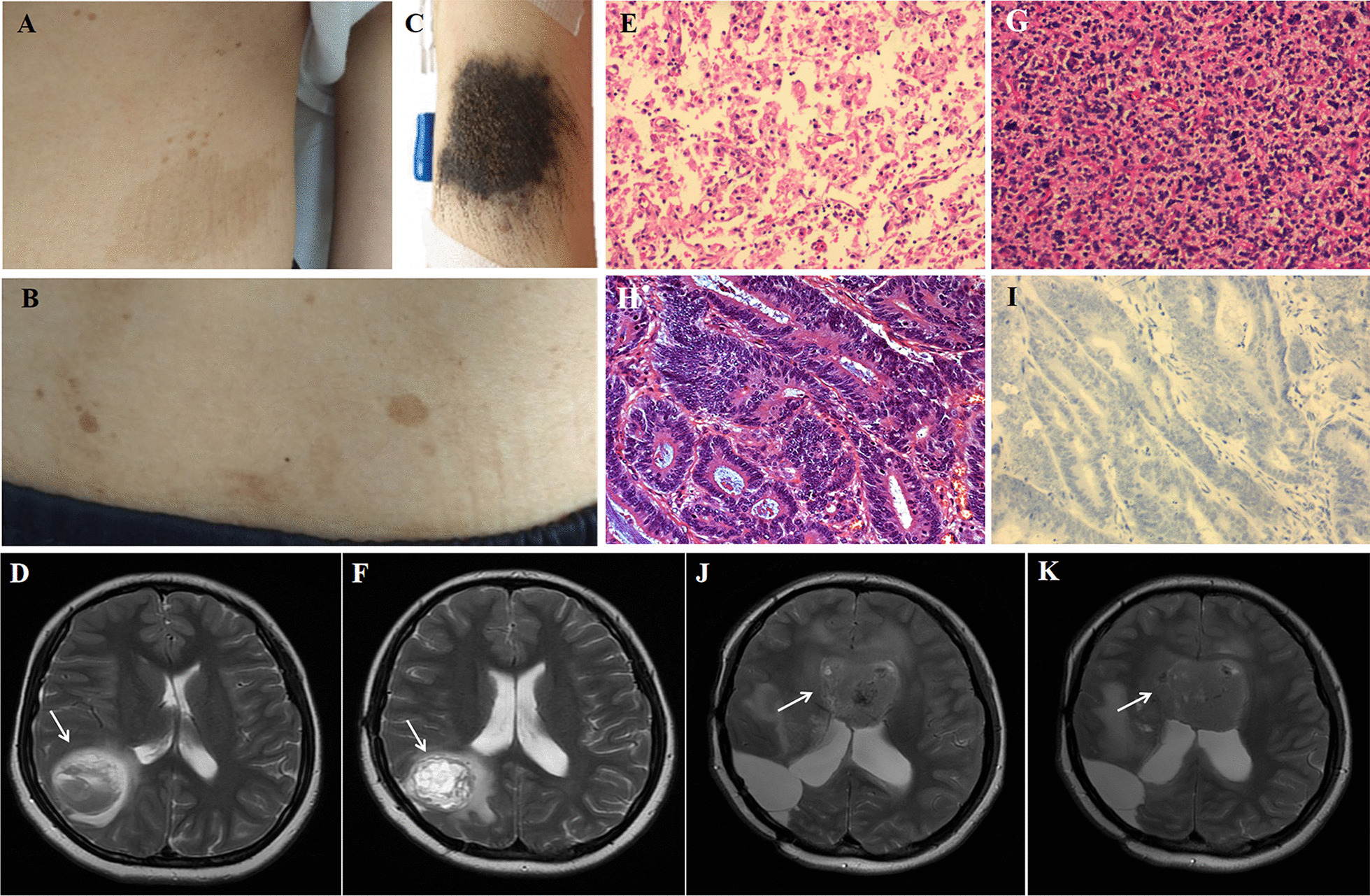
Photograph of the patient skin cafe´-au-lait macules located on **(A)** the anterior abdominal wall and **(B)** lower back and **(C)** a hairy pigmented cutaneous nevi located on the forearm. Pathologic findings showing **(E)** diffuse tumor cells, abundant focal cells with atypia, and vascular hyperplasia (HE, ×200); **(G)** diffuse tumor cells with different forms, multinucleated tumor cells, and focal interstitial small vessel hyperplasia (HE, ×200); **(H)** colon tubular adenomas, atypical tumor cells, deep-dyed big nucleolus and the mitosis were common (HE, ×200); **(I)** the expression loss of PMS2 proteins in both tumor and non-neoplastic tissue (IHC, ×200). MRI of the patient brain showing **(D)** a right temporal-occipital ring-enhancing lesion (arrow) of about 3.5 × 4.5 cm in size at the age of 16, **(F)** vascular enhancement of nodular mass (arrow) at the site of the previous surgery at the age of 16, **(J, K)** aggressive tumor relapse (arrow) at the age of 18

An 11-year-old girl was admitted to our hospital because of a polyp mass dropping out of the anus. Physical examination revealed multiple CALMs, mainly localized on the anterior abdominal wall (Fig. 1A) and lower back (Fig. 1B). The CAL lesions showed foci of skin hypopigmentation with irregular edges and within areas of dense freckles. She also had a hairy pigmented cutaneous nevi on her right forearm (Fig. 1C). Colonoscopy revealed two large polyps in rectus and anus. The pathological results of endoscopically-resected specimen were tubular adenomas with low-grade dysplasia. At the age of 16 she had a severe headache and brain MRI showed a right temporal-occipital ring-enhancing lesion of about 3.5 × 4.5 cm in size (Fig. 1D). She then underwent the gross total resection of the tumor and the pathological analysis demonstrated a glioblastoma multiforme (WHO grade II) and partial anaplastic astrocytomas (WHO grade III) (Fig. 1E). Given the suspected diagnosis of CMMRD, sequencing analysis of MMR genes was performed on both tumor and blood specimens. Other genes associated with childhood tumors were also analyzed, including *TP53*, *BRAF*, *PTCH1*, *NF1*, and *APC*, etc. The sequencing on genomic DNA demonstrated a homozygous deletion NM_00535.5:c.1577delA (p.Asp526fs) in exon 11 of the *PMS2* gene (Table 1). All other exons and their flanking regions showed a normal sequence. Molecular testing for MSI was also performed on tumor tissue using the Promega MSI analysis system v1.2, which included fluorescently labeled primers for co-amplification of five mononucleotide repeat markers (BAT-25, BAT -26, NR-21, NR-24 and MONO-27). The result showed high-frequency microsatellite instability (MSI-H). Four months after the first surgery, MRI revealed vascular enhancement of nodular mass at the site of the previous surgery which suggested tumor relapse (Fig. 1F). She underwent second surgical resection of the tumor (Fig. 1G), followed by cranial radiotherapy and temozolomide chemotherapy based on the Children's Oncology Group study [2]. She tolerated radiation therapy and temozolomide very well. However, when she started on maintenance cycles of oral temozolomide, she had severe pneumonia. Serum lymphocyte subset analysis showed a decrease in the absolute numbers of CD4+ T cells. Determination of serum immunoglobulin showed her IgG, IgA and IgM levels were profoundly decreased 1.36 g/L (normal 7–16), 0.258 g/L (normal 0.7–4), and 0.29 g/L (normal 0.4–2.3), respectively. She was forced to stop temozolomide treatment due to possible immunodeficiency and IgG substitution was given subsequently for the treatment of severe lung infection. At the age of 17 she did the second colonoscopy which revealed multifocal CR cancer lesions (2 in the transverse colon and 1 in the rectum). The patient then underwent subtotal proctocolectomy and the pathological results showed moderately differentiated CRC (T3N1M0) (Fig. 1H). Immunohistochemistry (IHC) staining detected expression loss of PMS2 protein in both CRC and non-neoplastic tissue (Fig. 1I), while MHL1, MSH2 and MSH6 were all expressed (Additional file 1: Fig. S1). Bevacizumab and topotecan were given due to metastatic CRC and gliomas. The MRI finding suggested an aggressive tumor relapse (Fig. 1J, K) and the patient died of the exacerbation of neurological symptoms at the age of 18.

**Table 1:** Genomic DNA sequencing of the patient and her family’s lymphocyte tissue

Patient and her family	Age (years)	Genotype (mutated gene, position, mutation at protein level, mutation at cDNA level)	Homozygous/heterozygous condition	RS number	Polyphen2 score	Minor allele frequency
Patient	16	*PMS2 *6026819, p.Asp526fs, c.1577delA	Homozygous condition	–	–	–
Mother	45	*PMS2* 6036980 G>C, p.S260S, c.780 C>G	Heterozygous condition	rs1805319	–	0.83127
		*PMS2* 6043386 G>A, p.A96A, c.288 C>T	Heterozygous condition	rs12532895	–	0.11362
Younger brother	5	*PMS2* 6036980 G>C, p.S260S, c.780 C>G	Homozygous condition	rs1805319	–	0.83127
		*PMS2* 6043386 G>A, p.A96A, c.288 C>T	Heterozygous condition	rs12532895	–	0.11362
		*PMS2* 6026775 T>C, p.K541E, c.1621 A>G	Homozygous condition	rs2228006	Benign (0.000)	0.88319
		*PMS2* 6045627 C>T, p.R20Q, c.59 G>A	Heterozygous condition	rs10254120	Benign (0.164)	0.07568

Genomic DNA sequencing of the patient’s lymphocyte tissue showed *PMS2* mutation NM_00535.5:c.1577delA (p.Asp526fs) in exon 11. Her mother and younger brother also carried *PMS2* mutations

## Discussion and conclusions

The rarity of CMMRD and its diverse tumor features pose both a diagnostic and managerial challenge. Our patient was first diagnosed as adenomatous polyps with low-grade dysplasia. Based on retrospective research on 288 pediatric patients with coloscopic-proved polyps, isolated juvenile polyps were the most frequent histopathological type while juvenile polyposis syndrome, Peutz-Jeghers syndrome, familial adenomatosis polyposis (FAP), and other cancer predisposition syndromes accounted for the rest kinds of tumorous polyps [3]. In fact, the condition that a pediatric patient was diagnosed with adenomatous polyps other than juvenile polyps presented a necessity for evaluation for inherited polyposis syndrome. More importantly, the CALMs found were highly reminiscent of an accompanying genetic condition. The most common condition associated with CALMs is neurofibromatosis type 1 (NF-1), which also has other features, such as skin-fold freckling, iris Lisch nodules, cutaneous and subcutaneous neurofibromas, and importantly, CNS gliomas [4]. CMMRD patients may present with other diagnostic features of NF-1, and these overlapped phenotypes lead to misdiagnosis of CMMRD as NF-1 if only based on clinical examination. Given that CALMs are the most common non-neoplastic manifestation of CMMRD, and the coupling polyps containing adenomatous change are considered major cancer precursor lesions, attention should be paid to the MMR defect. Unfortunately, the diagnosis was not made till the occurrence of brain gliomas because of the lack of a fully developed polyposis phenotype.

Gliomas, especially high-grade gliomas, constitute the largest proportion of brain tumors. Others include supratentorial primitive neuroectodermal tumors and medulloblastomas. Followed by the current consensus suggesting a genetic analysis be performed in the case of high-grade tumors including gliomas in the pediatric groups, sequencing analysis of MMR genes was done which demonstrated a novel homozygous loss-of-function mutation NM_00535.5:c.1577delA (p.Asp526fs) in *PMS2*. In the process of detecting homozygous mutation of the *PMS2* gene, we intercepted the 100 bp on both sides of the mutation site and analyzed them by high-throughput sequencing. The result showed it was inconsistent with the *PMS2CL*, the homologous pseudogene of *PMS2*, indicating that the mutation is not *PMS2CL* [5]. Considering that the patient had gliomas and CRC with MSI-H at a very young age, this frameshift mutation was deemed a driver mutation in her carcinogenesis and was the most likely cause of the phenotype. The cancer spectrum in CMMRD appeared to be related to the MMR gene mutated [6]. *PMS2* mutations are most frequent in CMMRD and responsible for almost 60% of cases, followed by *MLH1*, *MSH6* and *MSH2*. Patients having homozygous conditions in *PMS2* develop brain tumors within their first decade of life [6]. The identification of homozygous *PMS2* mutation status led to a necessity of gene surveillance of familial members. Two variants of the *PMS2* gene, S260S and A96A, were found not only in the patient’s mother aged 45 but the younger brother aged 5. These variants are nucleotide exchanges that are synonymous at protein level, meaning they do not cause changes of amino acids and have no apparent effect on the encoded protein. The patient’s brother also had two missense variants, K541E and R20Q, which were analyzed by Polyphen2 and predicted to be benign with a score of 0.000 and 0.164, respectively (Table 1). Also, two different in silico databases (ClinVar and SNPs&GO) consistently predicted that the known variants K541E and R20Q are not associated with MMR and LS. The patient’s mother and younger brother had no signs of malignancies or colorectal polyps, but the younger brother with two missense variants in the *PMS2* had multiple CALMs, so close surveillances including radiological, endoscopic and hematological screening have been recommended.

**Fig. 2:**
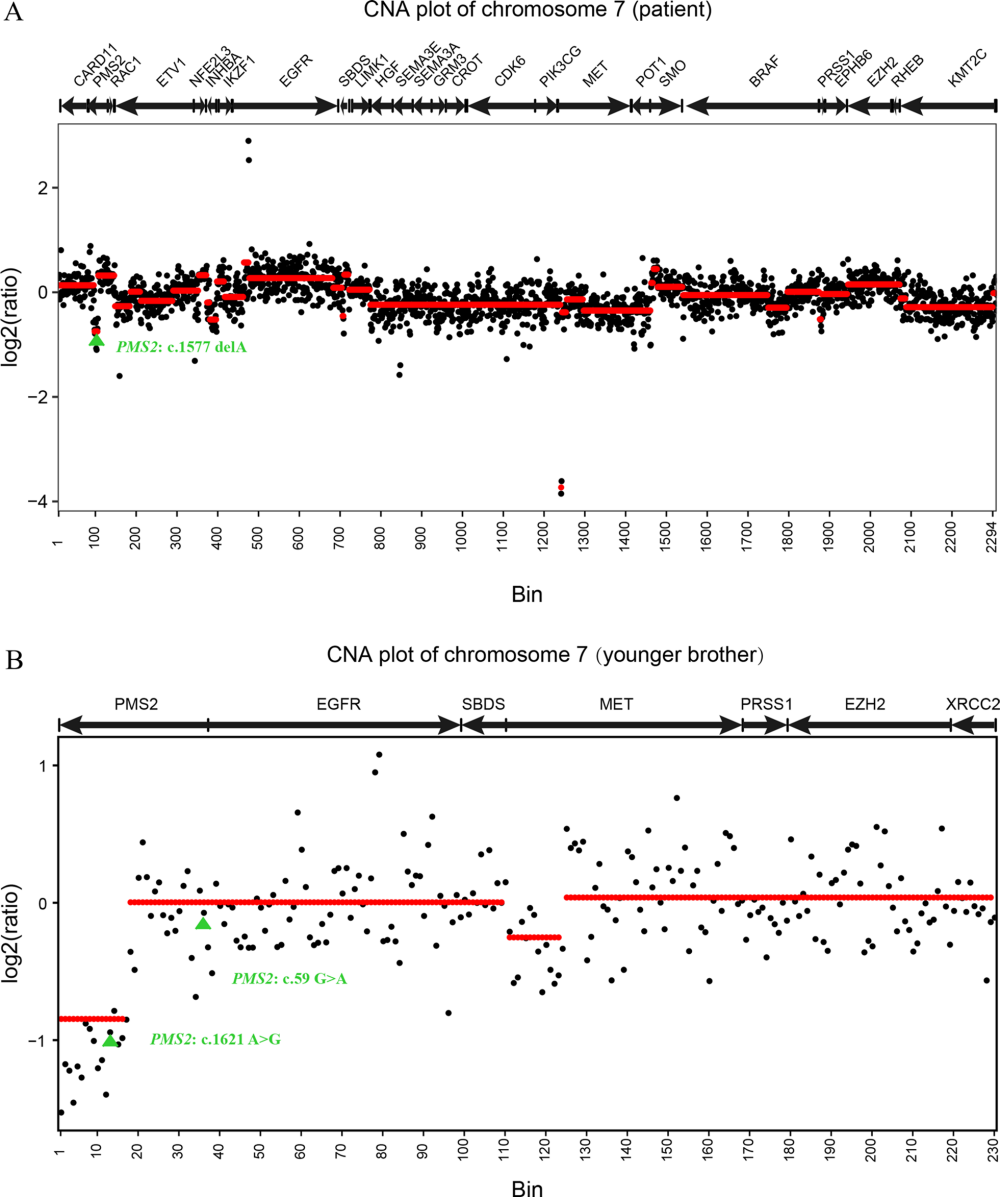
Germline copy number aberrations (CNAs) and LOH events in the patient and younger brother. The *PMS2* c.1577delA resides in the patient's LOH genomic region (**A**), and c.1621A>G and c.59G>A are located in the younger brother's LOH and diploid regions, respectively (**B**)

Intriguingly and rarely, this patient is homozygous for mutation p.Asp526fs but the mother is not a carrier of this mutation. And the father died of acute myocardial infarction at the age of 44, and there is no detailed data on whether her father had LS, in which LS patients frequently develop colorectal cancer before the age of 50 (average age at onset of disease: 45 years) [7]. After the maternity confirmed, we have performed germline copy number aberrations (CNAs) analysis and determined that the loss of heterozygosity (LOH) has occurred newly in the patient. Sequencing data (FASTQ) of WBC samples collected from 68 healthy individuals were used to construct a "reference panel", which underwent the same experiment and analysis procedures to ensure that it was comparable to the patient's WBC sample. For each reference sample, targeted genomic regions on chromosome 7 were binned into 100 bp. We then applied Circular Binary Segmentation (CBS) on depth ratio between the patient sample and reference. The results showed that c.1577delA resides in the patient's LOH genomic region (Fig. 2A), and c.1621A>G and c.59G>A are located in the younger brother's LOH and diploid regions, respectively (Fig. 2B). These present the diagnostic challenges and difficulties in identifying familial *PMS2* mutations.

Close colonoscopic surveillance has shown vast advantages by decreasing the modality and mortality of CRC for patients with cancer-prone polyposis syndromes [8]. Generally, colonoscopic screening recommendations depend on risk stratification including the age when colorectum polyps develop, the interval at which polyps grow, the frequency and rate at which polyps may progress to cancer [8]. Those with likely or confirmed hereditary CRC syndromes belong to high-risk patients and should initiate screening earlier and undergo surveillance at shorter intervals than average or increased risk individuals. For CMMRD patients, due to the faster progression of polyp to cancer development, colonoscopy is recommended beginning at the first decade of life or earlier and with a shorter screening interval of 6 months [1]. Video-capsule endoscopy and double-balloon enteroscopy are also recommended to identify small bowel adenomas or cancers. Unfortunately, the patient didn’t comply with surveillance protocols until the development of left-side CRC. Except for the delay in making CMMRD diagnosis, her reluctance to undergo interval screening was the main reason.

Current knowledge of therapeutic strategies and their outcomes in CMMRD are limited because the related information comes from limited research of individual patients or small patient series. Due to the high mutation rate caused by diminished replication repair, rapid onset and frequent recurrence of cancers may occur despite standard-of-care treatment. Chemotherapy remains a frontline treatment based on various phenotypes of CMMRD cancer, but effective chemotherapy drugs are still lacking. This is due to severe drug toxicity in children, MMR deficient associated resistance to chemotherapeutics, and the ability to accelerate somatic mutations which cause an increased risk of secondary tumors. Recently, there is growing evidence to support immunotherapy in the treatment of CMMRD cancers. Immune checkpoint inhibitors, such as programmed cell death-1 blockers, have been found to produce a successful clinical response in CMMRD patients holding MSI-H/hypermutated tumors [9]. Hypermutated tumors lead to the production of truncated protein products termed neoantigens. The preliminary efficacy of neoantigen-based vaccines has been observed in LS patients that show neoantigen-specific immune responses [10]. This creates fair expectations on this class of agents as an effective therapeutic approach for CMMRD. More importantly, given that intensive surveillance may not guarantee detection at a curable stage, preventive treatment strategy represents a promising avenue for CMMRD management. Aspirin has been shown potential in CMMRD patients for chemoprevention against MSI-H CRC, ovarian cancer, and T cell lymphoma [11]. The underlying mechanism is explained by nitric oxide-mediated apoptosis of cells of an MSI phenotype while further clarity is required [12].

Taken together, the diagnosis and treatments of a Chinese girl with CMMRD were outlined and the newly found mutation in *PMS2* will update our understanding of genomic variants which contributes to enhanced patient management. Importantly, for pediatric patients with CR adenomas, especially those with CALMs, we recommended the integration of molecular (or genomic) testing into routine clinical care to identify CMMRD.

## Supplementary Information

**Additional file 1. Fig. S1**: Positive immunohistochemistry for (A) MHL1, (B) MSH2 and (C) MSH6 in both tumor and non-neoplastic tissue (IHC, ×200).

## Data Availability

The datasets generated during the current study are available from the China National Genomics Data Center https://ngdc.cncb.ac.cn/gsa-human/browse/HRA000532, and are also available from the corresponding author on reasonable request. The contact person is Li Ying, email: yingli0209@163.com. The below listed databases were used for data analysis (weblinks provided). PolyPhen2: http://genetics.bwh.harvard.edu/pph2/; ClinVar: https://www.ncbi.nlm.nih.gov/clinvar/; SNPs&GO: https://snps-and-go.biocomp.unibo.it/cgi-bin/snps-and-go/runpred.cgi.

## Abbreviations

CMMRD: Constitutional mismatch repair deficiency
CALMs: Café-au-lait macules
CRC: Colorectal cancer
LS: Lynch syndrome
MMR: Mismatch repair
MSI-H: High-frequency microsatellite instability
NF-1: Neurofibromatosis type 1
HE: Hematoxylin and eosin staining
IHC: Immunohistochemistry
LOH: Loss of heterozygosity
CNAs: Copy number aberrations
