# Supplementary figures and images for: Diagnostic challenges in a CMMRD patient with a novel mutation in the PMS2 gene: a case report
Source: BMC Med Genomics. 2021 Jul 12;14:184. doi: 10.1186/s12920-021-01031-9 (PMC8274000; doi:10.1186/s12920-021-01031-9)

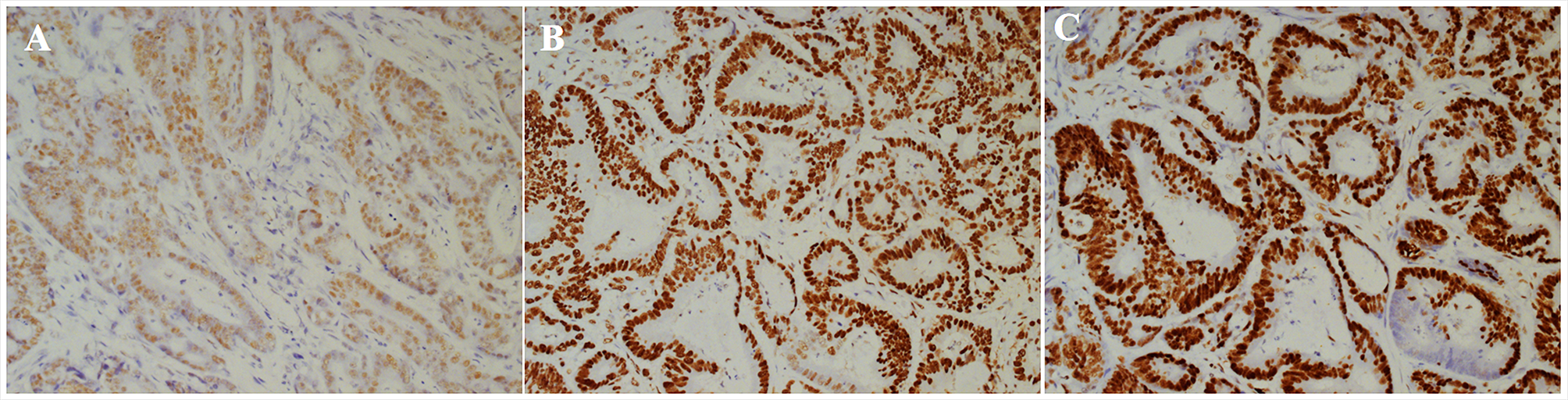

Supplement: Supplementary file 1 — Additional file 1. Fig. S1: Positive immunohistochemistry for (A) MHL1, (B) MSH2 and (C) MSH6 in both tumor and non-neoplastic tissue (IHC, ×200). [file 12920_2021_1031_MOESM1_ESM.tif]
